# The Bone-Forming Effects of HIF-1α-Transduced BMSCs Promote Osseointegration with Dental Implant in Canine Mandible

**DOI:** 10.1371/journal.pone.0032355

**Published:** 2012-03-05

**Authors:** Duohong Zou, Jiacai He, Kai Zhang, JieWen Dai, Wenjie Zhang, Shaoyi Wang, Jian Zhou, Yuanliang Huang, Zhiyuan Zhang, Xinquan Jiang

**Affiliations:** 1 Ninth People's Hospital Affiliated with Shanghai Jiao Tong University, School of Medicine, Shanghai Key Laboratory of Stomatology, Shanghai, China; 2 Department of Oral and Maxillofacial Surgery, School of Stomatology, Stomatological Hospital, Anhui Medical University, Hefei, China; 3 Department of Stomatology, First Affiliated Hospital of Bengbu Medical College, Anhui, China; 4 Department of Stomatology, Shanghai East Hospital Affiliated with Tongji University, Shanghai, China; University of Southern California, United States of America

## Abstract

The presence of insufficient bone volume remains a major clinical problem for dental implant placement to restore the oral function. Gene-transduced stem cells provide a promising approach for inducing bone regeneration and enhancing osseointegration in dental implants with tissue engineering technology. Our previous studies have demonstrated that the hypoxia-inducible factor-1α (HIF-1α) promotes osteogenesis in rat bone mesenchymal stem cells (BMSCs). In this study, the function of HIF-1α was validated for the first time in a preclinical large animal canine model in term of its ability to promote new bone formation in defects around implants as well as the osseointegration between tissue-engineered bone and dental implants. A lentiviral vector was constructed with the constitutively active form of HIF-1α (cHIF). The ectopic bone formation was evaluated in nude mice. The therapeutic potential of HIF-1α-overexpressing canine BMSCs in bone repair was evaluated in mesi-implant defects of immediate post-extraction implants in the canine mandible. HIF-1α mediated canine BMSCs significantly promoted new bone formation both subcutaneously and in mesi-implant defects, including increased bone volume, bone mineral density, trabecular thickness, and trabecular bone volume fraction. Furthermore, osseointegration was significantly enhanced by HIF-1α-overexpressing canine BMSCs. This study provides an important experimental evidence in a preclinical large animal model concerning to the potential applications of HIF-1α in promoting new bone formation as well as the osseointegration of immediate implantation for oral function restoration.

## Introduction

The immediate dental implant possesses several advantages over the delayed implant, including agonistic bone resorption post-extraction, reduced time required to make dentures, and immediate satisfaction with the aesthetics and function, especially at locations of formerly missing teeth [1]. Therefore, immediate post-extraction implants have high clinical value. However, the main challenge of immediate post-extraction implants is significant alveolar bone loss (height or width of alveoli) owing to periodontal disease, traumatic injury, congenital abnormalities, tumors, or physiological bone resorption. Due to autologous bone grafts limited clinical applications [2], [3], gene-enhanced tissue engineering method is attempted to promote bone repair and tissue regeneration, especially for challenging defect sites, where spontaneous repair is not achievable [4].

Bone mesenchymal stem cells (BMSCs) are multipotent stem cells that have the capacity to differentiate into cartilage, bone, fat and endothelial cells [5]; therefore, BMSCs are considered ideal seed cells to repair damaged tissue in a tissue engineering approach. A pivotal mechanism for BMSCs to promote tissue regeneration is by secreting various growth factors [6]. The combination of stem cell and gene therapies could be an optimal clinical strategy for tissue replacement/repair, where BMSCs are genetically modified to express higher levels of some specific factors. Growth factor-overexpressing stem cells have the potential to accelerate osteogenesis and angiogenesis in bone defects with tissue engineering technology; these growth factors include bone morphogenic protein (BMP), vascular endothelial growth factor (VEGF), and basic fibroblast growth factor [7], [8].

Compared with the above genes, the hypoxia-inducible factor-1α (HIF-1α) has many advantages in local gene therapy methods because of its effects as an upstream protein in the promotion of osteogenesis and angiogenesis [9], [10]. Our previous studies demonstrated that HIF-1α can enhance osteogenic expression in rat BMSCs both *in vitro* and *in vivo* [11]. To further investigate the value of HIF-1α in potential clinical applications, the current study sought to determine whether HIF-1α induces canine BMSCs to regenerate bone in a canine model of dental implant defects and making oral function restoration.

Because of excellent biocompatibility and osteoconduction, calcium phosphate biomaterials have been widely used as scaffold materials in pre-clinical studies [12]. Calcium-magnesium phosphate cement (CMPC) exhibits good biocompatibility, biodegradability and mechanical properties [13].

On the basis of the above data, we sought to use a combination of HIF-1α mediated canine BMSCs and CMPC scaffolds to repair mesi-implant defects in the canine mandible. In this paper, we tested the hypothesis that HIF-1α gene therapy promotes osseointegration between tissue- engineered bone and dental implants in a canine model.

## Results

### Characterization of BMSCs and gene transduction

**Figure 1:**
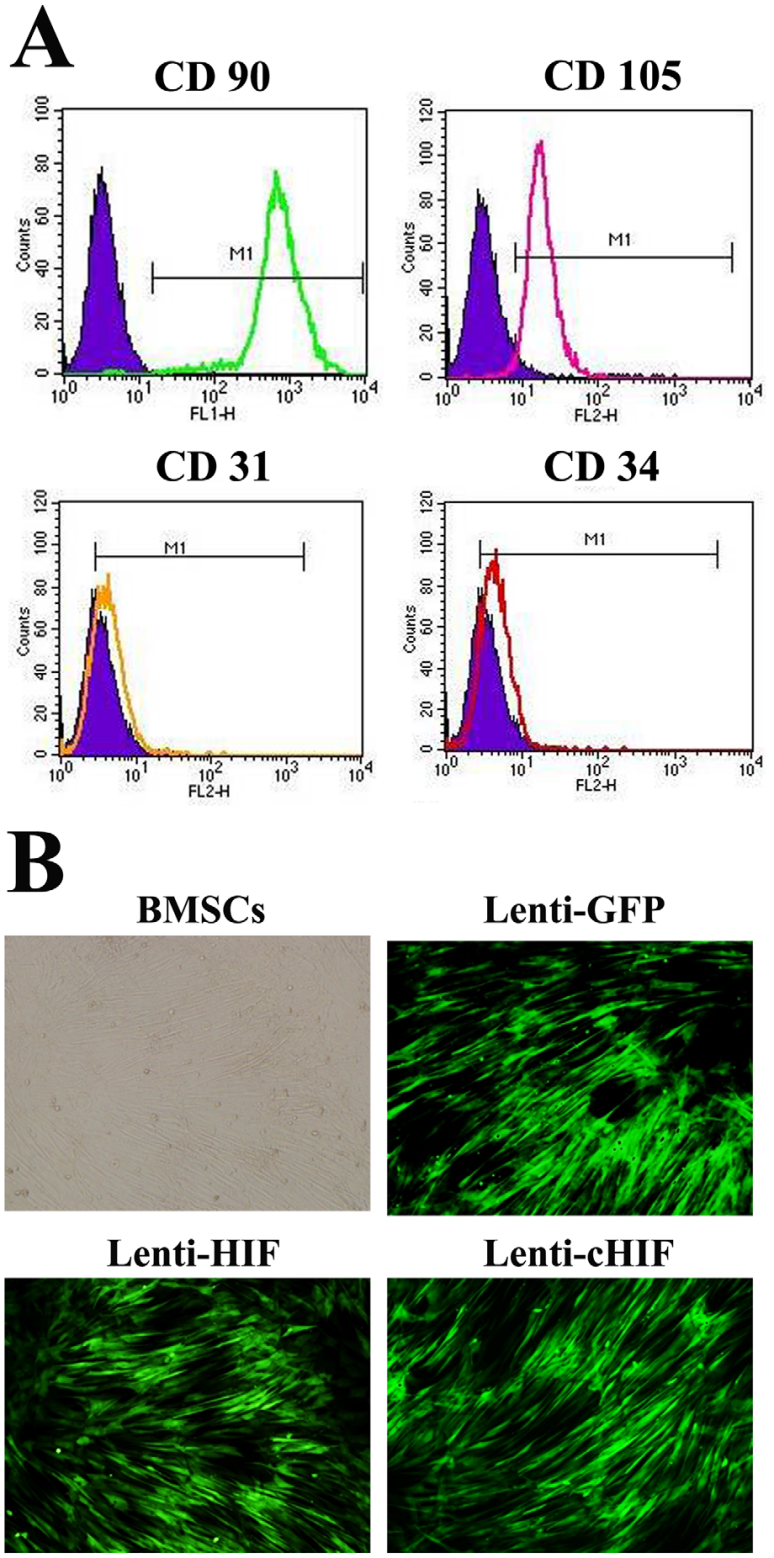
Characterization of F344 canine BMSCs and target gene transduction. Flow cytometry analysis of cell surface markers CD90, CD105, CD31, and CD34 (A); A multiplicity of infection of 7 pfu/cell achieved high transfer efficiency, around 90%, 4 days after Lenti-GFP, Lenti-HIF, and cHIF transduction of canine BMSCs (100×) (B).

**Figure 2:**
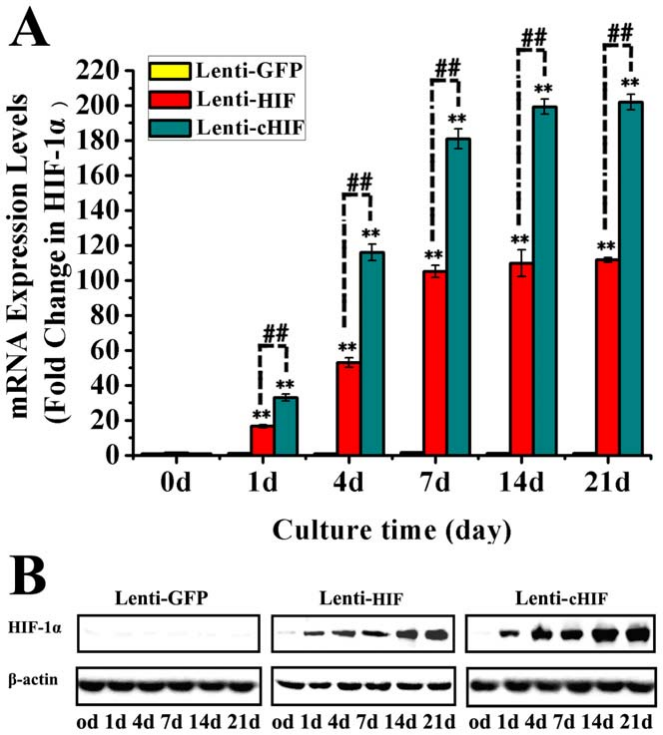
HIF-1α expression in canine BMSCs after target gene transduction. mRNA and protein expression levels in the canine BMSCs transduced by Lenti-HIF, Lenti-cHIF and Lenti-GFP On days 0, 1, 4, 7, 14, and 21 (A and B).

At passage 3, BMSCs were characterized by flow cytometry. CD90 and CD105 were highly expressed, whereas CD31 and CD34 were rarely detected (Figure 1A). Under an optimal multiplicity of infection (MOI = 7), BMSCs were transduced by Lenti-GFP, Lenti-HIF, and Lenti-cHIF. Four days after transduction, BMSCs fluoresced green under inverted fluorescence microscopy, showing efficiency of transduction of approximately 90% (Figure 1B). HIF-1α mRNA and protein expression was up-regulated in the target gene groups compared with the control group by qPCR and western blotting, respectively (Figure 2).

### Analysis of ectopic osteogenesis in nude mice

**Figure 3:**
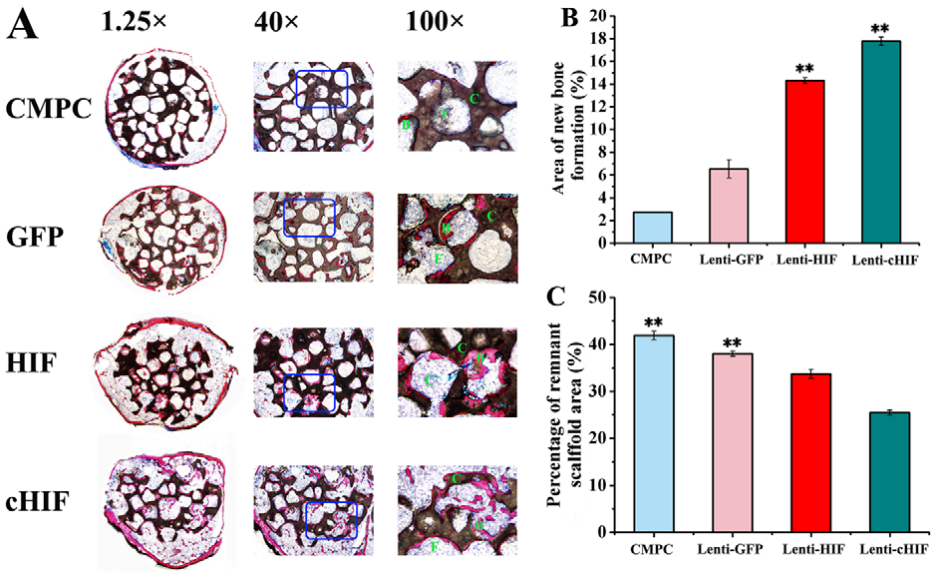
Observation of subcutaneous ectopic osteogenesis in the nude mice. (A) The undecalcified specimens were stained with van Gieson's picrofuchsin. From top to bottom: CMPC construct, Lenti-GFP- transduced BMSC/CMPC construct, Lenti-HIF-transduced BMSC/CMPC construct, and Lenti-cHIF-transduced BMSC/CMPC construct (F = fibroblastic-like tissue, C = CMPC, B = new bone; 1.25×, 40×, and 100×). (B). New bone formation area per 100× field in histological sections. (C) The percentage of remnant scaffold area per 100× field in histological sections. (**, *P*<0.01, the target gene groups compared to the GFP group or the CMPC group).

To determine whether HIF-1α-expressing BMSCs had the ability to promote ectopic osteogenesis *in vivo*, nude mice were subcutaneously implanted with HIF-1α constructs. Eight weeks after implantation in the subcutaneous tissue of nude mice, samples were excised. Their histology was analyzed under light microscopy (Figure 3A). The area of new bone formation was 17.89±1.69% of the total area per 100× field in the cHIF group, 14.25±1.21% in the HIF group, 6.63±1.53% in the GFP group, and 2.86±0.23% in the CMPC-only group (Figure 3B). The percentage of remnant scaffold area was 42.75%±2.62% of the total area per 100× field, 38.16%±1.92%, 34.21±3.14%, and 25.82%±1.98% in the CMPC, GFP, HIF, and cHIF groups, respectively (Figure 3C). These results demonstrate that constitutively active HIF-1α in BMSCs increases bone formation when incorporated with CMPC scaffolds.

**Figure 4:**
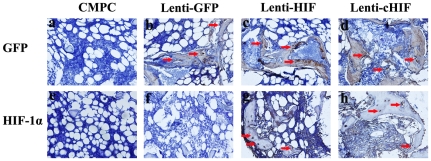
Immunohistochemical analysis of new bone formation in each group in the subcutaneous nude mice. Immunostaining for GFP of (a) CMPC group, (b) Lenti-GFP group, (c) Lenti-HIF group, and (d) Lenti-cHIF group. The GFP, HIF, and cHIF groups show positive brown staining in fibroblastic-like tissue and bone matrix (red arrow). The strong HIF-1α expression was stained in both the bone and the surrounding fibroblastic-like tissue (red arrows) in (g) Lenti-HIF group and (h) Lenti-cHIF group. There was no obvious positive staining in (e) the CMPC group or (f) the Lenti-GFP group (a–h, 400×).

To investigate the presence of the implanted BMSCs in the subcutaneous tissue of nude mice, GFP immunohistochemistry was performed. All lentivirus vehicles encoded EGFP. GFP was apparent in the new bone matrix or fibrous tissue in the HIF-, cHIF- and GFP-transduced BMSC groups 8 weeks post-operation, while the CMPC group showed negative staining (Figure 4a–d). Moreover, HIF-1α staining was found in the target gene-transduced groups in both the bone matrix and the surrounding fibroblast-like tissue, whereas there was no evident positive expression for endogenous HIF-1α in the Lenti-GFP-transduced BMSCs or the CMPC-alone group (Figure 4e–h).

### Comparative bone formation and osseointegration in bone defects of canine mandibles with simultaneous dental implantation

**Figure 5:**
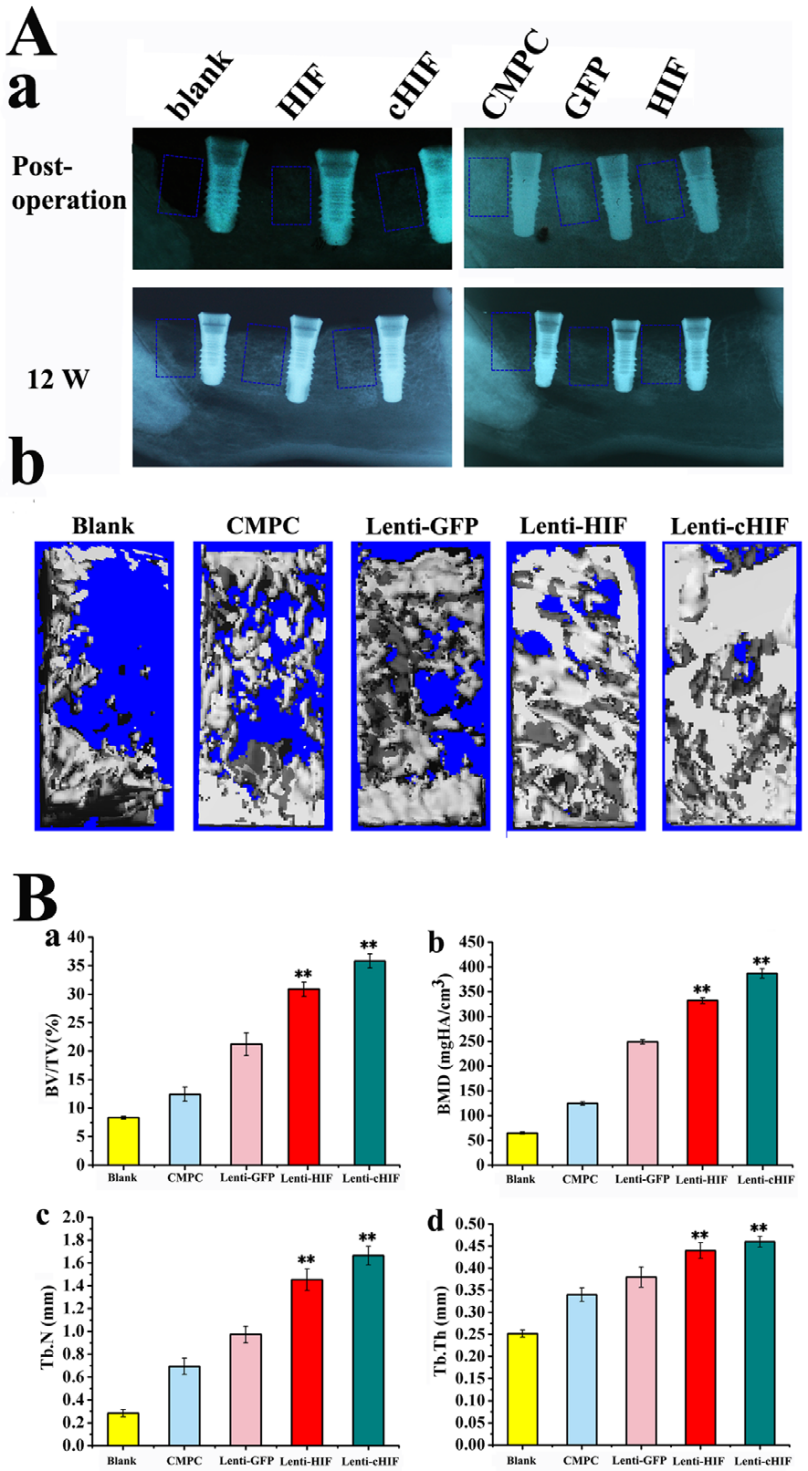
Radiography and micro-CT evaluation of bone repair and osseointegration at 12 weeks after implantation. X-ray images were taken immediately after surgery and at 12 weeks (A-a). The morphology of the newly formed bone in the defects was reconstructed using micro-CT (A-b). Morphometric analysis of the BV/TV (B-a), BMD (B-b), Tb.N (B-c), and Tb.Th (B-d). (**, *P*<0.01, the target gene groups compared to the blank group, the GFP group or the CMPC group).

To determine whether HIF-1α and CMPC scaffolds were able to promote bone formation and osseointegration, we used the various scaffolds constructs in a relevant canine model of dental implant defect. X-ray images were taken after surgery to observe the position of the filled materials in the defects. Radiography showed that scaffold materials were implanted in the correct position and tightly contacted the implant (Figure 5A-a). After 12 weeks, the radiographic evidence of new bone formation and osseointegration varied among the five groups. In the HIF-1α-expressing groups, new bone formation and osseointegration were superior to the GFP, CMPC and blank groups as measured by bone density and the bone contact ratio of dental implants (Figure 5A-a). The morphology of the newly formed bone in the defects was reconstructed using micro-CT, which showed that the new bone formation in the HIF and cHIF groups was greater than that in the other groups at 12 weeks post-operation (Figure 5A-b). Morphometrical measures were used to calculate the amount of newly formed bone in the defect sites. The calculated parameters showed that BV/TV in the Lenti-HIF and Lenti-cHIF groups was significantly greater than that in the GFP group or CMPC group (Figure 5B-c). Additionally, BMD, Tb.N, and Tb.Th were similarly increased in the target gene-transduced groups (Figure 5B-c and d).

**Figure 6:**
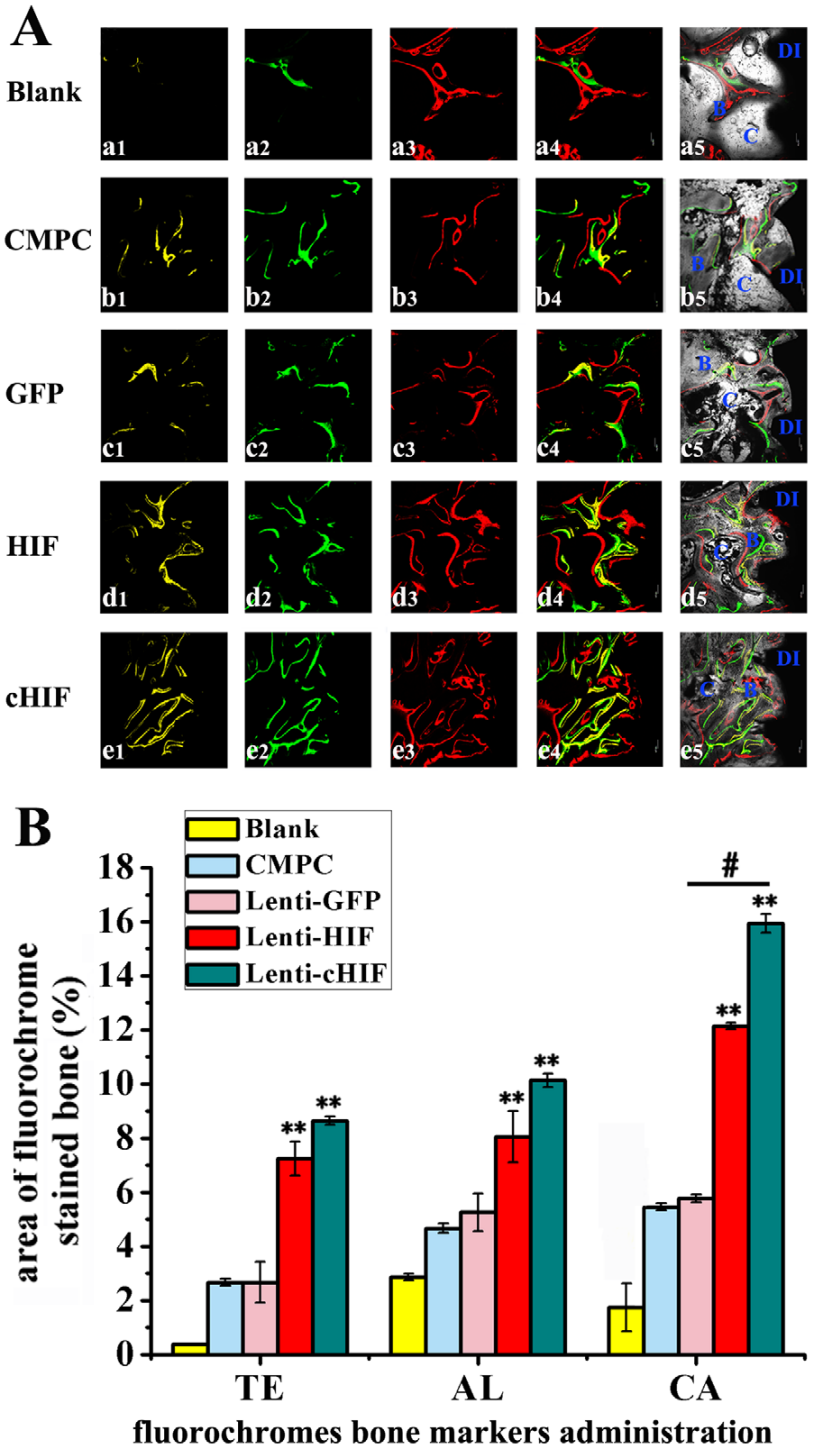
New bone formation and mineralization determined histomorphometrically by TE, CA, and AL fluorescent quantification, which represent the mineralization level at 12 weeks after the operation (A). Parts a, b, c, d, and e are confocal laser scanning microscopy images for each group. Parts a4, b4, c4, d4, and e4 represent merged images of the three fluorochromes for the same group. Parts a5, b5, c5, d5, and e5 represent the merged images of the three fluorochromes together with the plain confocal laser microscopy image for the same group. (B) The graph shows the percentage of each fluorochrome area in each group. (** *P*<0.01, target gene groups compared with the GFP group, Blank group or CMPC group; #, *P*<0.05, the cHIF group compared to the HIF group).

Using sequential fluorescent labeling, bone mineralization and apposition were measured. At 1 week, the percentage of TE labeling in the HIF group was 7.11±0.74%, which was greater than the CMPC group (2.96±0.47%), the blank group (0.46±0.07%) or the GFP group (2.98±0.89%) (*P*<0.01), but less than the percentage in the cHIF group (8.87±0.14%) (Figure 6A-a1, b1, c1, d1, e1 and B). At 4 weeks, the percentage of CA labeling was 10.12±0.18%, 8.01±1.02%, 5.22±0.84%, 4.82±0.21%, and 2.96±0.11%, for cHIF, HIF, GFP, CMPC, and blank, respectively (Figure 6A-a2, b2, c2, d2, e2, and B). The cHIF group CA labeling was significantly different from GFP, CMPC and blank (*P*<0.01), and the HIF group CA labeling was significantly different from GFP, CMPC and blank (*P*<0.01), but there no significant difference between the cHIF and HIF groups (*P*>0.05) (Figure 6B). At 8 weeks, the percentage of AL labeling was 16.02±0.32%, 12.78±0.11%, 5.65±0.13%, 5.42±0.12%, and 1.83±1.12% for cHIF, HIF, GFP, CMPC, and blank, respectively (Figure 6A-a3, b3, c3, d3, e3, and B), with a significant difference between cHIF and HIF (*P*<0.05) (Figure 6B). Taken together, these results show that HIF-1α over-expression effectively enhances new bone formation and mineralization.

**Figure 7:**
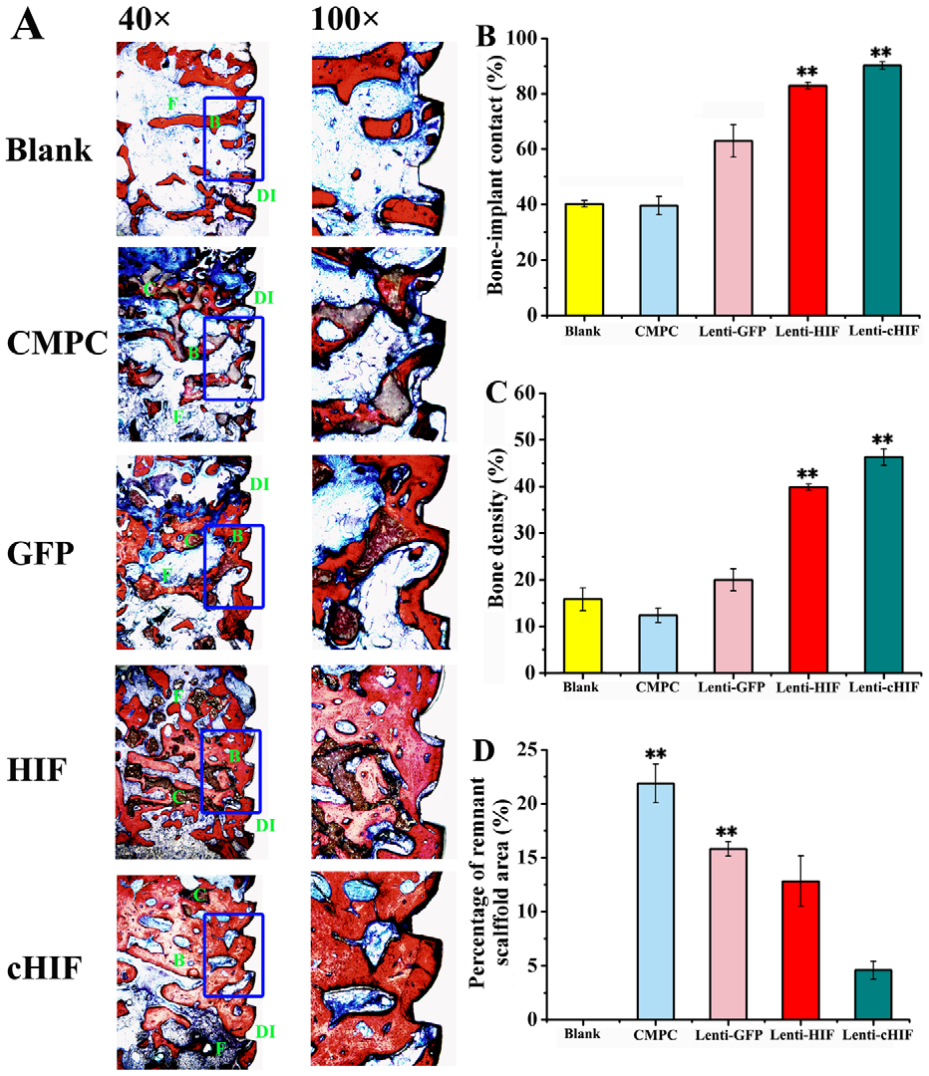
Histological analysis of newly formed bone and remnant scaffold area in calvarial defects. The specimens were sliced, and sections were stained with van Gieson's picrofuchsin. From top to bottom: Blank, CMPC construct, Lenti-GFP-transduced BMSCs/CMPC construct, Lenti-HIF-transduced BMSCs/CMPC construct, and Lenti-cHIF- transduced BMSCs/CMPC construct (F = fibroblastic-like tissue, C = CMPC, B = new bone, DI = dental implant; original magnification, 40×, 100×) (A). BIC per 40× field in histological sections (B). Bone density per 40× field in histological sections (C). The percentage of remnant scaffold area per 100× field in histological sections (D). (** *P*<0.01, target gene groups compared with the GFP group, Blank group or CMPC group).

To further investigate the HIF-1α-mediated functional restoration of tissue-engineered bones in a large animal, we evaluated bone repair and osseointegration in canines using histologic and histomorphometric methods (Figure 7A). Under light microscopy, BIC was 91.24%±2.12% of the total area per 40× field in the cHIF group, 83.57%±2.33% in the HIF group, 62.94%±6.62% in the GFP group, 38.96%±4.87% in the CMPC group, and 40.06%±1.88% in the blank group. BIC in each target gene groups was significantly higher than the control groups (*P*<0.01), and no significant difference was observed between the CMPC group and the blank group (Figure 7B) (*P*>0.05). The newly formed bone showed varying degrees of bone density. Bone density was 46.82±4.64% of the total area per 40× field in the cHIF group, 40.02±1.82% in the HIF group, 20.06±5.12% in the GFP group, 12.37±2.31% in the CMPC group, and 16.76±5.24% in the blank group. There were significant differences in bone density between the cHIF or HIF group and each control group (*P*<0.01), but no significant difference was seen among the three control groups (Figure 7C). Furthermore, the percentage of remnant scaffold area was measured. These percentages were 22.16%±5.53%, 15.95%±1.87%, 12.25±6.42%, and 4.97%±2.26% of the total area per 100× field in the CMPC, GFP, HIF, and cHIF groups, respectively (Figure 7D).

## Discussion

To simulate the clinical situation as closely possible, gene therapy or a tissue-engineered construct must be tested in a large animal model before it can be introduced in a clinical trial [14]. In this study, we detected the ability of HIF-1α to promote bone formation in defects around implants and stimulate osseointegration in the canine mandible.

Gene-enhanced tissue engineering is a promising strategy for bone regeneration. HIF-1α is a transcriptional activator that functions as a master regulator of responses to tissue ischemia and regulates many downstream target genes, including those involved in angiogenesis, red blood cell maturation, energy metabolism, and cell proliferation and viability [15], [16]. However, HIF-1α is rapidly degraded under normoxic conditions. A HIF-1α truncation/substitution mutant (deletion of amino acids 392–520 and the substitutions Pro567Thr and Pro658Gln) and a point mutant (proline 564 to alanine, proline 402 to alanine and aminosuccinic acid 803 to alanine) both remain stable and active in normoxic conditions [11], [17]. The truncated form of HIF-1α shows greater stability and activity *in vitro* under non-hypoxic conditions compared with the point mutant HIF-1α and wild-type HIF-1α (HIF). Therefore, we transduced canine BMSCs with the truncated mutant allele of HIF-1α (cHIF) to increase osteogenesis via a tissue engineering approach. HIF-1α can enhance rat BMSC osteogenesis [11], [17]. However, there have been no reports on bone formation by using HIF-1α-mediated stem cell therapy in large animal models. It was unclear whether HIF-1α also promotes the osteogenesis of canine BMSCs.

To investigate whether HIF-1α promotes canine BMSCs osteogenesis *in vivo*, subcutaneous ectopic osteogenesis was examined in nude mice. We observed abundant new bone formation and regenerated tissue with a greater area of new bone and lesser remnant scaffold in the target gene groups. Histomorphometric parameters showed that HIF-1α significantly enhanced canine BMSCs osteogenesis in these nude mice, and the cHIF group induced superior effects compare to the HIF group. Additionally, immunohistochemistry demonstrated the presence of the implanted canine BMSCs and the expression of HIF-1α in these new bone areas. These results provide a good basis for future studies to detect bone repair defects in canine models.

The immediate implant possesses advantages over delayed implants, such as preventing bone resorption, saving the time of making dentures, and obtaining immediate aesthetic results [1]. However, the presence of insufficient bone volume is a challenge for the correct implantation of dental implants in the jaw. In this study, 12 weeks post-operation, X-ray images and micro-CT revealed that HIF- and cHIF-transduced cells repaired nearly the whole defect area. Additionally, good osseointegration was obtained between tissue-engineered bone and dental implants in the target gene groups. More intensive bone formation was seen in the cHIF group than in the HIF group, whereas only minor repairs were observed in the GFP and CMPC groups. Furthermore, the quantitative analysis by micro-CT showed more newly formed bone in the cHIF group than in the HIF, GFP, and GS groups according to BMD, BV/TV, Tb.N, and Tb.Th. The bone mineralization and apposition in bone defects, as indicated by fluorescent labeling, were consistent with the morphometric findings. Histological examination demonstrated that BIC and bone density in the cHIF group were superior to the other groups. BIC is an evaluation of expected and actual bone contact on machined and osseotite implant surfaces [18]. BIC exerts an important effect on the success of dental implants, such as in terms of implant loss, the weight-bearing ability of dentures, and the duration of implant survival in the oral cavity. The above data provided reliable base that cHIF can promote osseointegration in the bone defects. Of course, a long-term follow-up for the new bone formation *in vivo* must be considered if we use HIF-1α as a means of clinical treatment in the future.

Many groups have investigated potential therapies of ischemic disease with HIF-1α in rat models [19]–[21]. HIF-1α gene-based therapy can repair critical-sized calvarial defects in rats [11], [17]. However, to our knowledge, there have been no reports about the use of HIF-1α in repairing bone defects for oral function restoration in large animal models. This study provides a basis for future investigations of HIF-1α-transduced BMSCs in repairing bone defects. However, a major concern in gene-mediated stem cell therapy is safety. In our study, we found no evidence that the HIF-1α-overexpressing canine BMSCs formed tumors 12 weeks *in vivo*. Furthermore, Rajagopalan et al. have used HIF-1α to treat the lower extremity of patients with critical limb ischemia [22]. But a prolonged observation *in vivo* will still be required to confirm the safety of HIF-1α-transduced BMSCs in humans.

In summary, this study confirms that HIF-1α-transduced canine BMSCs significantly promoted new bone formation both subcutaneously (ectopically) and in defects around implants in canine mandibles *in vivo*. Furthermore, osseointegration between tissue-engineered bone and dental implants was enhanced by HIF-1α. This work may contribute to potential clinical applications of BMSCs transduced with HIF-1α for augmenting the bone mass of the jaw and oral function restoration.

## Materials and Methods

### Ethics Statement

The Ethics Committee for Animal Research at the Ninth People's Hospital affiliated to Shanghai Jiao Tong University approved all the experimental protocols involving the use of dogs and nude mice.

### Animals

Five adult male Labrador retrievers approximately 1.5 years old, each weighing 25.0–35.0 kg, were used in the immediate dental implant experiment. Six male nude mice approximately 6–8 weeks old were used in the subcutaneous ectopic osteogenesis experiment. The dogs were all given the same soft diet under the standard laboratory conditions, and the nude mice were fed a daily pellet diet in specific pathogen-free surroundings.

### BMSCs culture and gene transduction

After each dog was anesthetized by ketamine (10 mg/kg) and xylazine (4 mg/kg), 5 mL of autologous bone marrow was drawn by needle aspiration from the iliac crests. The BMSCs were cultured in Dulbecco's Modified Eagle Medium (DMEM) (Gibco BRL, Grand Island, NY, USA) supplemented with 10% fetal bovine serum (FBS, HyClone, Logan, UT, USA) and 1% penicillin/streptomycin at 37°C in 5% CO_2_. After 5–7 days, the culture solution was removed, and fresh medium was added. When the BMSCs reached 80–90% confluence, the cells were detached with trypsin/EDTA (0.25% w/v trypsin, 0.02% EDTA) and then transferred to 10 cm dishes at a concentration of 1.0×10^5^ cells/mL in 10 mL of medium. The culture medium was then changed every 3 days, and the cells were used for all experiments at passage 2–4. The BMSCs were characterized by flow cytometry for CD90, CD105, CD31, and CD34 expression (Invitrogen, Carlsbad, CA, USA).

A replication-defective lentivirus that encoded enhanced green fluorescent protein (EGFP) was used as the vector for this study. Lenti-GFP (the control group, only GFP protein), Lenti-HIF (wild-type HIF-1α, target gene group), and Lenti-cHIF (a constitutively active form of HIF-1α, target gene group) were constructed as previously described [11]. The optimum efficiency of lentiviral gene transfer in BMSCs was a multiplicity of infection (MOI) of 7. The transduction efficiency was assessed by counting the number of GFP-positive cells after 4 d of culture under the inverted microscope. After BMSCs was transduced with Lenti-cHIF, Lenti-HIF, or Lenti-GFP, target gene was detected on days 0, 1, 4, 7, 14, and 21 with RT-qPCR and western blotting analysis, as previously described [11] (Table S1).

### Preparation of BMSCs/CMPC constructs

CMPC scaffolds (cylinders, Φ 5 mm×2 mm^3^) were supplied by East China University of Science and Technology, Shanghai, China. The scaffolds were sterilized using ^60^Co irradiation. The CMPC had an open porosity of 75% and an average pore diameter of 400 µm±50 µm. After cells were detached from culture dishes, BMSCs (2.0×10^5^ cells/mL) in suspension were gently added to the scaffolds until saturation. The BMSCs/CMPC constructs were incubated for an additional 4 h to allow for cell attachment before use. The constructs were used as described below. BMSCs/CMPC constructs were obtained after 24 h incubation and then characterized by scanning electron microscopy (Philips SEM XL-30, Amsterdam, Netherlands) (Figure S1 a and b).

### Subcutaneous ectopic osteogenesis in nude mice

Six nude mice were anesthetized by intraperitoneal injection of pentobarbital (Nembutal 3.5 mg/100 g). The groups of Lenti-cHIF/BMSCs/CMPC, Lenti-HIF/BMSCs/CMPC, Lenti-GFP/BMSCs/CMPC, and CMPC constructs were bilaterally implanted subcutaneously into the backs of nude mice. The 12 sides of 6 nude mice were randomly divided into four groups, with 3 sides per group. After 8 weeks of growth, specimens were harvested.

### Dental implantation in the canine mandible

The surgical procedures were performed on Labrador retrievers. During immediate implantation, the dogs were anesthetized by intramuscular injection of ketamine (10 mg/kg) and xylazine (4 mg/kg). Under sterile conditions, the premolars (from the first to the fourth) of the mandible were extracted with a tungsten-carbide bur forceps. The model of dental implant defect was completed as previously reported [23]. Briefly, after detachment from the gum, the alveolar bone was completely exposed. Six mesial bone defects (both sides of the mandible) adjacent to the mesial socket at a 6 mm height, 5 mm in the mesio-distal direction, and 4 mm in the bucco-lingual direction were created (Figure S1 c). Then, the Ø3.5×12 mm implants (Cowellmedi Atlas Implant Inc., Korea) were installed into the bone defects. After the primary stability of the implant was obtained, the cover screw was added. Five dogs with 30 defects were generated and randomly allocated into the following graft study groups: (1) blank (n = 6), (2) CMPC (n = 6), (3) CMPC/BMSCs/Lenti-GFP (n = 6), (4) CMPC/BMSCs/Lenti-HIF (n = 6), and (5) CMPC/BMSCs/Lenti- cHIF (n = 6). The mesial bone defects were filled with the constructs from the bottom to the top in direct contact with the implant, except in the blank group (unfilled). When the sulcular flaps were adapted to eliminate tension, the incision was closed with interrupted and horizontal mattress sutures (Figure S1 d-k). The first week after surgery, the animals received amoxicillin (500 mg, twice daily) and ibuprofen (600 mg three times daily) via the systemic route. The dogs were checked daily and fed soft food. To detect the degree and quantity of new bone, the dogs were intraperitoneally administered by hydrochloride tetracycline (TE, Sigma, 25 mg/kg), calcein (CA, Sigma, 20 mg/kg), and ARS (AL, Sigma, 30 mg/kg) at 1, 4, and 8 weeks post- operation, respectively [24]. All animals were sacrificed 12 weeks after dental implant insertion.

### Radiography and micro-CT measurement

Under general anesthesia, X-ray images were taken to detect the scaffold-filled bone defects as well as the new bone formation and mineralization at after-operation and 12 weeks post-operation. Radiographs were taken by a dental X-ray machine (Trophy, France). The morphology of the reconstructed mandible was assessed using an animal micro-CT scanner (eXplore Locus SP, GE Healthcare, Milwaukee, UK). Briefly, the specimens were scanned with the following parameters: 14 µm resolution, 210° rotation angle, 0.4° incremental rotation angle incremental, 80 kV voltage, 80 µA current, 2960 ms exposure time, frame average of 4 pixel combinations for 1×1, 80 kV X-ray tube potential, 80 µA tube current, and 14-µm voxel resolution. After the micro-CT scan, the visualization of bone was processed with the following image parameters: 16.897 mm×13.442×6.474 mm original image size, 29 µm image resolution, and 16 image digits. Micro-CT measurements in the bone defect included the bone volume to total bone volume ratio (BV/TV), bone mineral density (BMD), the trabecular number (Tb.N), and the trabecular thickness (Tb.Th).

### Histological and histomorphometric observation

The composites of nude mice were harvested 8 weeks post-operation. The undecalcified specimens of 6 nude mice (4 specimens from each group) were processed following these steps: (1) dehydration in a graded series of ethanol from 75% to 100%; (2) embedding in polymethylmethacrylate (PMMA); (3) sectioning (150 mm thick) using a microtome (Leica, Hamburg, Germany); (4) polishing the sections to a final thickness of approximately 40 mm; and (5) staining the sections with Van Gieson's picrofuchsin [25]. Lastly, under an inverted microscope, sections were observed for new bone formation and remnant scaffolds. To detect the presence of the implanted BMSCs in subcutaneous nude mouse sites, other specimens of 6 nude mice (5 specimens from each group) were decalcified in 10% EDTA for 3 weeks. Immunohistochemistry was performed as previously reported [11]. Briefly, the tissue slides were processed by dewaxing, rehydration, and quenching endogenous peroxidase activity. The slides were mounted, and primary antibodies against GFP and HIF-1α (1∶1000 dilution) (Abcam, Inc., Cambridge, UK) were applied to the sections at 4°C overnight. Secondary antibody (Boster Co. Ltd, Shanghai, China) was applied to the slides for 30 min at room temperature. Then, the slides were developed with DAB substrate (DAKO, Cambridge, UK) and finally counterstained with hematoxylin.

For immediate dental implantation in the mandible in canines, all specimens were embedded in PMMA and prepared with undecalcified sections. To analyze mineralization in the bone defect area, the fluorescent labeling of sections was observed using a confocal laser scanning microscope (CLSM) (Leica TCS Sp2 AOBS, Heidelberg, Germany). After sections were stained with Van Gieson's picrofuchsin, bone-implant contact (BIC), bone density (BD) and remnant scaffold were quantified using a computer-based image analysis system (Image Pro 5.0, Media Cybernetic, Silver Springs, MD, USA) as previously described [26]. In each case, the BIC, which was calculated as (total length of bone contact/total length of implant surface)×100%, of the whole length of the implant in the bone defect area was measured, except for the coronal part and the apical part of the dental implant. The BD, which was calculated as (total surface of bone in the reference area/total reference area)×100%, of the whole bone defect area was measured, except for the coronal part and the apical part. The remnant scaffold was measured as a percentage of the section per 100× field.

### Statistical analysis

Data are expressed as the mean ± SD. Using the SAS 6.12 statistical software package (Cary, NC, USA), statistical significance was assessed by an ANOVA with Tukey's post-hoc test. *P*<0.05 was considered statistically significant (* *P*<0.05 and ** *P*<0.01, target gene (HIF or cHIF) groups compared with the control group; # *P*<0.05 and ## *P*<0.01, the cHIF group compared with the HIF group).

## Supporting Information

Figure S1 **Scanning electron microscopic evaluation of the CMPC microstructure and surgical procedure.** 48 h after seeding, the cells have spread well on the surface of the scaffold (a and b). Diagram of the mesi-dental implant defects in canine mandible: a 6 mm height, 5 mm in the mesio-distal direction, and 4 mm in the bucco-lingual direction (c). Making bone defects with a 4-mm-diameter trephine bur (d and e). Installing dental implants in the tooth socket (f–h). The graft study groups were allocated (CMPC, CMPC/BMSCs/Lenti-GFP, CMPC/BMSCs/Lenti-HIF, and CMPC/BMSCs/Lenti-cHIF) (i and j). Closing the incision (k).
(TIFF)

Table S1 **A series of data, including the gene names, accession numbers, primer sequences, and amplicon sizes, is listed.**
(DOC)

## References

[1] Boix D, Gauthier O, Guicheux J, Pilet P, Weiss P (2004). Alveolar bone regeneration for immediate implant placement using an injectable bone substitute: an experimental study in dogs.. J Periodontol.

[2] Sjostrom M, Sennerby L, Nilson H, Lundgren S (2007). Reconstruction of the atrophic edentulous maxilla with free iliac crest grafts and implants: a 3-year report of a prospective clinical study.. Clin Implant Dent Relat Res.

[3] Joshi A (2004). An investigation of post-operative morbidity following chin graft surgery.. Br Dent J.

[4] Kaigler D, Avila G, Wisner-Lynch L, Nevins ML, Nevins M (2011). Platelet-derived growth factor applications in periodontal and peri-implant bone regeneration.. Expert Opin Biol Ther.

[5] Salinas CN, Anseth KS (2009). Mesenchymal stem cells for craniofacial tissue regeneration: designing hydrogel delivery vehicles.. J Dent Res.

[6] Fierro FA, Kalomoiris S, Sondergaard CS, Nolta JA (2011). Effects on proliferation and differentiation of multipotent bone marrow stromal cells engineered to express growth factors for combined cell and gene therapy.. Stem Cells.

[7] Xiao C, Zhou H, Liu G, Zhang P, Fu Y (2011). Bone marrow stromal cells with a combined expression of BMP-2 and VEGF-165 enhanced bone regeneration.. Biomed Mater.

[8] Qu D, Li J, Li Y, Gao Y, Zuo Y (2011). Angiogenesis and osteogenesis enhanced by bFGF ex vivo gene therapy for bone tissue engineering in reconstruction of calvarial defects.. J Biomed Mater Res A.

[9] Wang V, Davis DA, Haque M, Huang LE, Yarchoan R (2005). Differential gene up-regulation by hypoxia-inducible factor-1alpha and hypoxia-inducible factor-2alpha in HEK293T cells.. Cancer Res.

[10] Maynard MA, Evans AJ, Hosomi T, Hara S, Jewett MA (2005). Human HIF-3alpha4 is a dominant-negative regulator of HIF-1 and is down-regulated in renal cell carcinoma.. FASEB J.

[11] Zou D, Zhang Z, He J, Zhu S, Wang S (2011). Repairing critical-sized calvarial defects with BMSCs modified by a constitutively active form of hypoxia-inducible factor-1alpha and a phosphate cement scaffold.. Biomaterials.

[12] Zhang C, Wang KZ, Qiang H, Tang YL, Li Q (2010). Angiopoiesis and bone regeneration via co-expression of the hVEGF and hBMP genes from an adeno-associated viral vector in vitro and in vivo.. Acta Pharmacol Sin.

[13] Ooms EM, Egglezos EA, Wolke JG, Jansen JA (2003). Soft-tissue response to injectable calcium phosphate cements.. Biomaterials.

[14] Buma P Schreurs W, Verdonschot N (2004). Skeletal tissue engineering-from in vitro studies to large animal models.. Biomaterials.

[15] Maxwell PH, Wiesener MS, Chang GW, Clifford SC, Vaux EC (1999). The tumour suppressor protein VHL targets hypoxia-inducible factors for oxygen-dependent proteolysis.. Nature.

[16] Semenza GL (2009). Regulation of oxygen homeostasis by hypoxia-inducible factor 1.. Physiology (Bethesda).

[17] Zou D, Zhang Z, Ye D, Tang A, Deng L (2011). Repair of critical-sized rat calvarial defects using genetically engineered bone marrow-derived mesenchymal stem cells overexpressing hypoxia-inducible factor-1alpha.. Stem Cells.

[18] Trisi P, Lazzara R, Rao W, Rebaudi A (2002). Bone-implant contact and bone quality: evaluation of expected and actual bone contact on machined and osseotite implant surfaces.. Int J Periodontics Restorative Dent.

[19] Sarkar K, Fox-Talbot K, Steenbergen C, Bosch-Marce M, Semenza GL (2009). Adenoviral transfer of HIF-1alpha enhances vascular responses to critical limb ischemia in diabetic mice.. Proc Natl Acad Sci U S A.

[20] Bosch-Marce M, Okuyama H, Wesley JB, Sarkar K, Kimura H (2007). Effects of aging and hypoxia-inducible factor-1 activity on angiogenic cell mobilization and recovery of perfusion after limb ischemia.. Circ Res.

[21] Rey S, Lee K, Wang CJ, Gupta K, Chen S (2009). Synergistic effect of HIF-1alpha gene therapy and HIF-1-activated bone marrow-derived angiogenic cells in a mouse model of limb ischemia.. Proc Natl Acad Sci U S A.

[22] Rajagopalan S, Olin J, Deitcher S, Pieczek A, Laird J (2007). Use of a constitutively active hypoxia-inducible factor-1alpha transgene as a therapeutic strategy in no-option critical limb ischemia patients: phase I dose-escalation experience.. Circulation.

[23] Zhang Y, Song J, Shi B, Wang Y, Chen X (2007). Combination of scaffold and adenovirus vectors expressing bone morphogenetic protein-7 for alveolar bone regeneration at dental implant defects.. Biomaterials.

[24] Pautke C, Vogt S, Tischer T, Wexel G, Deppe H (2005). Polychrome labeling of bone with seven different fluorochromes: enhancing fluorochrome discrimination by spectral image analysis.. Bone.

[25] Wang S, Zhao J, Zhang W, Ye D, Yu W (2011). Maintenance of phenotype and function of cryopreserved bone-derived cells.. Biomaterials.

[26] Yamada Y, Ueda M, Naiki T, Nagasaka T (2004). Tissue-engineered injectable bone regeneration for osseointegrated dental implants.. Clin Oral Implants Res.

