# Supplementary figures and images for: The Bone-Forming Effects of HIF-1α-Transduced BMSCs Promote Osseointegration with Dental Implant in Canine Mandible
Source: PLoS One. 2012 Mar 5;7(3):e32355. doi: 10.1371/journal.pone.0032355 (PMC3293808; doi:10.1371/journal.pone.0032355)

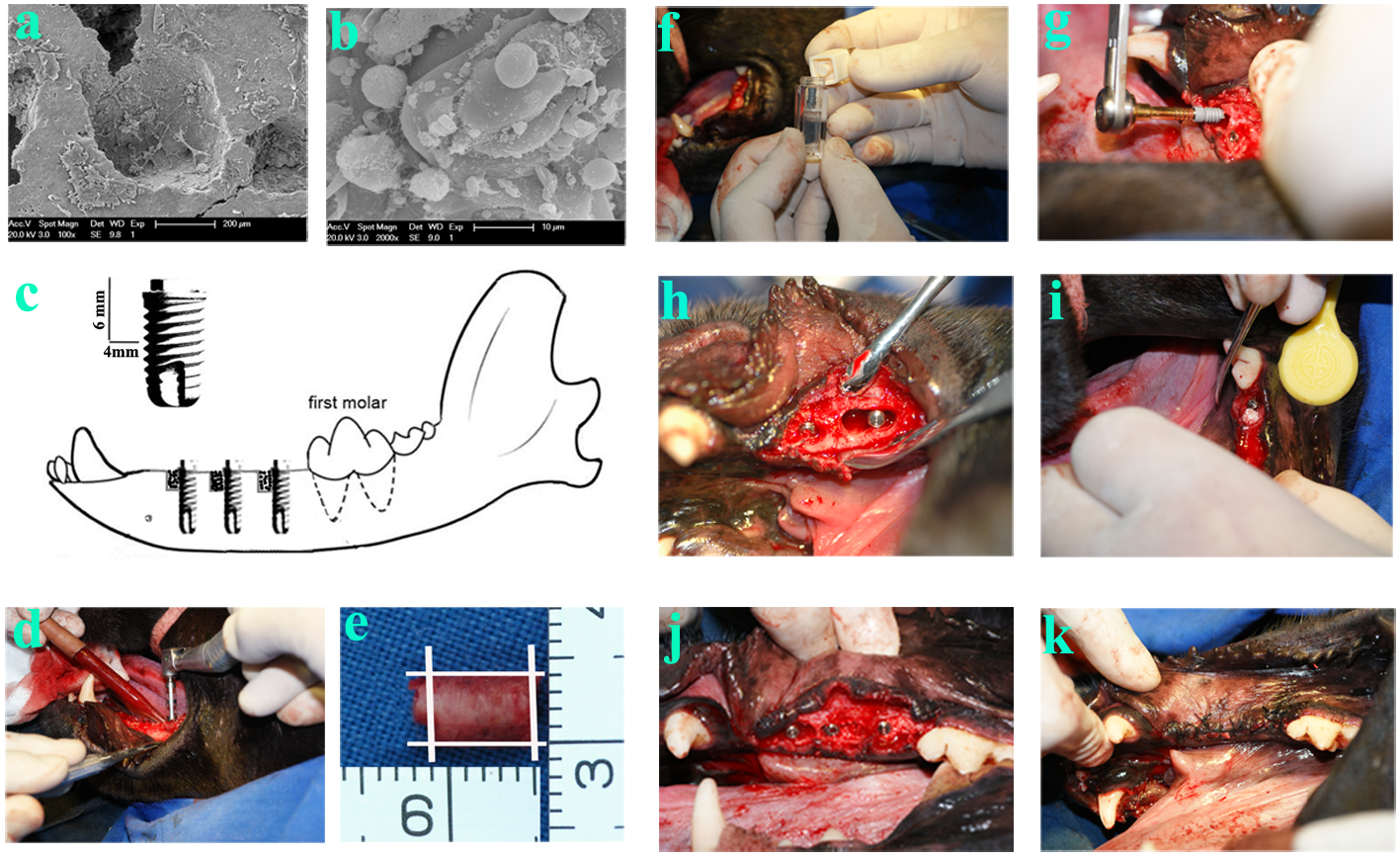

Supplement: Figure S1 — Scanning electron microscopic evaluation of the CMPC microstructure and surgical procedure. 48 h after seeding, the cells have spread well on the surface of the scaffold (a and b). Diagram of the mesi-dental implant defects in canine mandible: a 6 mm height, 5 mm in the mesio-distal direction, and 4 mm in the bucco-lingual direction (c). Making bone defects with a 4-mm-diameter trephine bur (d and e). Installing dental implants in the tooth socket (f–h). The graft study groups were allocated (CMPC, CMPC/BMSCs/Lenti-GFP, CMPC/BMSCs/Lenti-HIF, and CMPC/BMSCs/Lenti-cHIF) (i and j). Closing the incision (k). (TIFF) [file pone.0032355.s001.tiff]
