# Supplementary material for: The Bone-Forming Effects of HIF-1α-Transduced BMSCs Promote Osseointegration with Dental Implant in Canine Mandible
Source: PLoS One. 2012 Mar 5;7(3):e32355. doi: 10.1371/journal.pone.0032355 (PMC3293808; doi:10.1371/journal.pone.0032355)
Supplement: Table S1 — A series of data, including the gene names, accession numbers, primer sequences, and amplicon sizes, is listed. (DOC) [file pone.0032355.s002.doc]

Table 1

Nucleotide sequences for real-time RT-PCR primers.

| Genes | Primer sequence (5’-3’)  (forward/reverse) | Product size  (bp) | Annealing temperature (℃) | Accession  number |
| --- | --- | --- | --- | --- |
| HIF-1α | GTGTACCCTAACTAGCCGAGGA  GTTCACAAATCAGCACCAAGC | 149 | 60 | NM_001530.3 |
| GAPDH | ATGTTTGTGATGGGCGTGAAGGTCTTCTGGGTGGCAGTGAT | 174 | 60 | NM_017008.3 |
